# Home Vaccination

**Published:** 1898-12-17

**Authors:** 


### Home Vaccination.

The more carefully the Vaccination Act, and the order of the Local Government Board in accordance with which its provisions will have to be carried out are studied, the more does the whole business of vaccination seem to bristle with difficulties—difficulties for the most part connected with expense. Let us look at the matter of fees, dealing for the present merely with the vaccination of infants born in the district, which of course forms the main work of the public vaccinator. This official is to be paid (1) a minimum of one shilling for every child whose birth has been registered in his district, with the exception, however, of children who have died or have removed within four months of birth, and of children who have been certified to have been successfully vaccinated, or to be insusceptible, or whose parents have obtained a certificate of having satisfied the magistrate of their conscientious objection; (2) not less than five shillings for every successful primary vaccination performed at the child's home. It will thus be seen that the public vaccinator will receive a minimum of six shillings for each primary vaccination of an infant done at the child's home, together with a few stray shillings which he may receive for children who may die, or may remove between the age of four months and the time when he may receive a notice to vaccinate. The chief objections that have been raised by public vaccinators to these terms have to do with the large amount of work which will in many cases be involved in earning the fees named. Under no circumstances can six shillings for two visits be looked upon as good pay, when it is remembered that these visits cannot be made just when they happen to be convenient to the doctor, or when he happens to be passing, but must be made by previous appointment, and must take place within certain fixed hours; that a lot of book-keeping has to be done, and that if any illness results from the vaccination the after attendance on the case is included in the six shillings. To understand, however, what the fee is to cover, one must bear in mind that, according to his instructions, the public vaccinator must vaccinate only subjects who are in good health, and, in regard to infants, "must ascertain that there is not any febrile state, nor any irritation of the bowels, nor any unhealthy state of the skin, especially no chafing or eczema behind the ears, or in the groin, or elsewhere in folds of skin." Nor must he vaccinate where there has been recent exposure to infectious diseases. Nor is that all. He must not vaccinate if he thinks that the operation cannot be safely done "on account of the condition of the house," or because there has been "a recent prevalence of infectious disease in the district." So that, before the doctor can do the vaccination, he is expected to make a very complete examination of the infant, a sanitary investigation of the condition of the house, and an inquiry into the prevalence of epidemic diseases in the district. All this is included in the work of the first visit, the payment for which depends on successful vaccination, and all these things he must do "diligently" if he is to escape blame and possibly damages. If, then, the doctor discovers any one of the various conditions mentioned as contra indicating the operation, and gives a certificate authorising the postponement of the vaccination (which he has to do), and in addition sends a notification to the medical officer of health (as he is bound to do if it is the house that is in fault, or, if there is any prevalence of epidemic diseases), does he get any pay for all this work? Not a halfpenny beyond the original one shilling birth fee. Moreover, there is no knowing how often this process may have to be repeated, and for these further visits there will be no payment at all. Nothing beyond the one shilling birth fee is due until successful vaccination has been performed, and the practitioner has given the child such medical treatment as it shall, in his opinion, require in consequence. However many visits and examinations and reports he may make, the total six shillings is to cover it all; while if the child dies before the vaccination is done, or if it is removed into another district, or if in the end the parents refuse to have it vaccinated, however many visits he may have paid the vaccinator does but get the grand total of one shilling. That is for the town. But think of such an arrangement as this in country places, where a visit and back may mean a ride of a dozen miles. It is quite obvious that in view of the fact that notice of visits has to be sent, and that paid agitators are sure to stir people up to make the whole business as difficult as possible, as, for example, by being "not at home" when the doctor calls, the minimum fees appointed are likely to prove inadequate even in the towns, while for country districts they are manifestly absurd. The success of the Vaccination Act depends, then, upon the goodwill of the guardians, and on their readiness to spend a good deal more money upon it than the appointed minimum, and we doubt whether they will do it.

